# Assessing restaurant nutrition quality and dietary factors that influence purchase of food away from home across different food security levels

**DOI:** 10.3389/fpubh.2026.1727510

**Published:** 2026-01-23

**Authors:** Alyssa Anderson, Kiwon Lee, Natalie Caine-Bish, Elena Blaginykh

**Affiliations:** 1 School of Health Sciences, Kent State University, Kent, OH, United States; 2 School of Foundations, Leadership, and Administration, Kent State University, Kent, OH, United States; 3 College of Public Health, Kent State University, Kent, OH, United States

**Keywords:** dietary preferences, eating behavior, FAFH, food security, nutritional quality, restaurants

## Abstract

Both environmental and social factors influence the types and amount of food consumed away from home. To assess and understand the need for food and nutrition assistance programs targeting food consumed away from home, we investigated restaurants’ nutritional quality and community related dietary factors (i.e., dietary values and preferences) influencing food away from home consumption across neighborhood food security levels within an urban county in the Midwest. We found that restaurants in more food secure areas offered slightly healthier options and promoted healthy eating more than those in less secure areas. Participants living in both less food secure and more food secure neighborhoods valued taste, safety, and nutrition similarly. While those living in less food secure neighborhoods prioritized price more and perceived healthy food as too costly to eat, compared to those living in more food secure neighborhoods. These findings highlight the need for policy, systems, and environmental changes that address food consumed away from home.

## 1 Introduction

Food away from home (FAFH) spending in the U. S. has been exceeding food at home spending since 2010 and accounted for 58.5% of total food spending in 2023 (1). Data from NHANES (2002–2014) demonstrates FAFH frequency does not differ by food security level (2). The difference between FAFH purchasing practices lies in the types of food service establishments frequented; individuals with lower incomes tend to purchase FAFH at fast food venues while individuals with higher incomes tend to purchase from full-service restaurants (3). This difference may be attributable to several factors, including the higher density of fast-food outlets in low-income neighborhoods (4), differing expectations regarding food cost and quality (5), and greater demand for service attributes among higher-income consumers (6). Higher frequency of fast-food consumption has been linked to poor diet quality with higher energy intake (7). While food assistance programs provide food insecure individuals with food for at home preparation, many individuals continue to eat FAFH frequently due to various reasons (8). Therefore, restaurant food options and individual FAFH preferences are important to consider when addressing health concerns related to the food environment.

During 2023, 13.5% of population (47.4 million people) in the U. S. lived in food-insecure households (USDA, 2025), which is the highest rate over the past two decades except for the recession period (9). Experiencing food insecurity increases an individual’s risk of negative health outcomes and diet related chronic diseases (10) such as cardiovascular disease (11). Current research and policy efforts that aim to address the root causes of food insecurity and related health disparities focus on assessing and implementing changes within neighborhood food environments (12).

Public food assistance programs, such as the Supplemental Nutrition Assistance Program (SNAP) (13) and the Special Supplemental Nutrition Program for Women, Infants, and Children (WIC) (14), aim to improve the health status of those living in food-insecure households by increasing food access. Non-profit food assistance programs, such as food banks or Meals on Wheels, also contribute to reducing food insecurity (15). Current policies in place across these different food assistance resources focuses on increasing access to food prepared at home. For instance, both SNAP (13) and WIC (14) exclude the purchase of most convenience and hot food items. However, barriers to home food preparation, such as inadequate cooking facilities or appliances and lack of time, make it difficult to prepare meals at home in food insecure households (8). While some states have started addressing this issue by opting into the SNAP Restaurant Meals Program, it is not widely adapted across the United States and only certain SNAP clients are eligible to participate (16). Overall, FAFH purchases are rarely addressed by these resources and their policies despite its influence on overall diet quality.

This study investigates the following research questions: (1) What are the nutritional qualities of restaurant environments in more food secure versus less food secure neighborhoods? and (2) How do dietary factors related to FAFH consumption differ between more food secure and less food secure neighborhoods? Both food insecure and secure groups consume FAFH frequently (1, 2), but the quality of their FAFH consumption is known to differ (3, 7). The information gathered will help determine specific differences in dining environments between more food secure and less food secure neighborhoods and factors, such as frequency of eating FAFH, that influence eating behaviors of those living within these neighborhoods. The food environment has changed drastically in recent years, therefore, even though some of these factors may have been measured in previous research, it is important to consider them to determine any differences that may exist currently between these two groups. Ultimately, the information gained from this study can assist policymakers in developing effective assistance programs that promote a balanced diet.

## 2 Materials and methods

### 2.1 Study site

This study was conducted in an urban county within the midwestern United States where the median household income was $57,563, which was lower than the state ($65,070) and U.S. ($64,944) values (17). The percentage of families living below the poverty level was 11.9%, higher than the state (9.6%) and U.S. (9.1%) values (17). Overall, 17.1% of households had at least one of the following problems: overcrowding, high housing costs, lack of kitchen, or lack of plumbing facilities (17). Because there are differences in diet quality across the U.S. and with respect to the rural and urban environment, it was important to narrow the population studied to improve generalizability of similar populations (18). This county was selected based on feasibility criteria, including resource availability, variation of food insecurity levels by zip code, and logistical access.

When looking at food security across different neighborhoods within the county, the Food Insecurity Index (FII) ranks and compares the different zip codes (19). FII levels were calculated through a nationally available and validated tool that bases scores on factors related to different social determinants of health (19). FII scores ranged from 1 to 100, with higher scores indicating greater levels of food insecurity (19). Then, zip codes were ranked locally based on these numbers on a scale of 1–5, with 5 facing the lowest food security level (19). Within this county, 11 zip codes represented the lowest needs (FII level 1) and 11 zip codes represented the highest needs (FII level 5), out of a total of 51 zip codes (FII level 2: 10 zip codes, FII level 3: 7 zip codes, FII level 4: 12 zip codes).

### 2.2 Study design and data collection

This study was conducted in two phases. The objective of phase one was to answer the first research question by assessing the nutritional environment of local restaurants, and the objective of phase two was to answer the second research question by evaluating dietary factors that influenced consumption of restaurant food in more food secure or less food secure neighborhoods. Ethical approval for the study was obtained from the Office of Research Compliance at Kent State University.

The county health department provided the research team with a list of 6,682 licensed foodservice facilities. Closed establishments, schools, churches, hospitals, convalescent homes, bars or clubs with age limit for entry, movie theaters, museums, stores, mobile restaurants, workplace cafeterias, catering companies, and hotels were excluded from the study. After exclusion, a total of 2,363 restaurants remained. Each restaurant was then assigned a random digit code. Starting with the restaurant assigned “1,” the research team determined the level of the neighborhood FII based on the restaurant zip code. Assigned FII values ranged from 1 to 5 (17). This process continued until a balanced distribution was achieved across all levels of food security.

To measure the nutritional quality of restaurant environments, the Nutrition Environment Measures Survey for Restaurants (NEMS-R) was utilized (20). It consists of items that measure the availability of healthy items (e.g., whole grain bread, low-fat or fat-free salad dressing, or non-fried vegetables), as well as facilitators (e.g., reduced sized portions, nutrition information, or highlighting healthy options) and barriers (e.g., large portions encouraged, all-you-can-eat option, or unhealthy eating encouraged) to healthy eating. This reliable and valid tool has been adopted in research projects in various countries and often modified to accommodate study purposes and locations (21). This study modified several items in the original measurement instructions to improve clarity and better align with the research purposes: (1) ice cream stores were excluded from the specialty shops category, (2) sub/burger buns were included for whole grain bread options, (3) 2% milk was added to low-fat milk options, (4) the descriptors of baked, broiled, roasted, grilled, smoked, sautéed, stir-fried, steamed, boiled, poached, lean or extra lean, no breading or sauce, not fried, light or no mayo/sauce, broth based were considered for counting healthy entrees when nutrition information is not available, and (5) Caesar salad and house salad were considered as a main dish salad.

Researchers with NEMS-R experience trained the raters. As part of the training, raters assessed two restaurants using NEMS-R to practice and clarify any questions. Between July 2024 and January 2025, a pair of trained raters visited restaurants, introduced themselves, explained the study purpose, and completed assessments with the restaurants’ approval. A researcher thoroughly compared the two assessments for every restaurant, and any discrepancies were addressed by reviewing the raters’ comments on specific items. The final set of assessments included 18, 18, 18, 16, and 15 restaurants (totaling 85 restaurants) in FII levels 1, 2, 3, 4, and 5, respectively.

Different factors that influence consumption of restaurant foods and their frequency of eating FAFH were measured through a survey to gather aggregate data of people residing in neighborhoods that were more and less food secure. Recruitment occurred from August 2024 to February 2025 at various locations (46 sites total) throughout the county. These included: 7 restaurants (all restaurant site locations contacted for the NEMS-R assessment were invited), 27 branches of the county library system, 11 food pantries, and 1 farmer’s market. For this study, FII levels 1 and 2 are categorized as “more food secure neighborhoods.” FII levels 3, 4, and 5 are categorized as “less food secure neighborhoods.” The final set of survey recruitment locations included 9 sites in FII level 1 neighborhoods, 11 sites in FII level 2 neighborhoods, 15 sites in FII level 3 neighborhoods, 6 sites in FII level 4 neighborhoods, and 5 sites in FII level 5 neighborhoods.

To increase participation and accessibility, survey recruitment occurred through both flyers and in-person engagement. Participants were given the option of completing the survey via paper-pencil or online. The paper-pencil surveys were used to include participants that did not feel comfortable with using technology. In-person, paper-pencil surveys were completed at consenting restaurant assessment sites and a food pantry. Flyers with information on the study and a link to a Qualtrics survey were shared on site with the county library system, local food pantries, and a farmer’s market. In total, 212 surveys were completed by participants whose responses met inclusion criteria for analysis (18 years of age or older and live within targeted zip codes); forty-two surveys were completed by paper-pencil and 170 surveys were completed through Qualtrics. Participants were given the option to receive a $5 gift card after completing the survey.

Participants answered questions related to the frequency of eating out at fast food or full-service restaurants in a typical week. The importance of food taste, safety, price, and nutrition when selecting foods at restaurants was measured based on a 7-point Likert scale, following Lusk and Briggeman (22): 1 Not at all important to 7 Extremely important. Several items measuring dietary information were borrowed from FoodAPS (23) and are presented as follows: “It costs too much for (me/my family) to eat healthy foods” (agree/disagree), “I’m too busy to take the time to prepare healthy foods” (agree/disagree), and “I do not think healthy foods taste good” (agree/disagree). Additional demographic information such as home zip code, gender, ethnicity, race, and age was collected.

### 2.3 Data analysis

Restaurants and participants were grouped by their zip codes’ FII level (17). In this study, FII levels 1 and 2 were grouped together because they face the lowest needs related to food security and are labeled as “more food secure neighborhoods” in our study. FII levels 3, 4, and 5 face higher levels of need related to food security and are referred to as “less food secure neighborhoods.” This grouping mirrors the approach used by the Adult Food Security Survey Module when looking at food security based on two levels (24). The assessments of 36 restaurants in more food secure neighborhoods and 49 restaurants in less food secure neighborhoods were analyzed. A total of 212 participants completed the survey. Listwise deletion was used to exclude surveys that were missing data on key variables, resulting in 180 surveys for data analysis. Of these, 141 surveys were completed by participants residing in less food secure neighborhoods and 39 surveys were completed by participants residing in more food secure neighborhoods.

Using the NEMS-R scoring system, the nutritional quality of restaurants was calculated by awarding points for the availability of healthful options and facilitators of healthy eating and deducting points for barriers to healthy eating (Range: −5 to 21). Since the Kolmogorov–Smirnov and Shapiro–Wilk tests rejected the normality assumption, scores were compared between the two FII groups using the Kruskal-Wallis test. Adoption rates of individual assessment items at restaurants were compared between the two groups using Chi-Square tests.

Mann–Whitney U tests were used to determine differences in FAFH visits between residents of more food secure neighborhoods and less food secure neighborhoods as well as the importance of food taste, safety, and nutrition when selecting foods at restaurants. Mann–Whitney U was selected due to Kolomogorov-Smirnov and Shapiro–Wilk Tests rejected the normality assumption. Chi-Square test was used to determine if there is an association between food security groups when groups were asked about cost and if they have a time constraint to eating healthy.

## 3 Results

### 3.1 Characteristics of assessed restaurants

The types of assessed restaurants were similarly distributed as follows (Table 1): fast food (37.6%), sit-down (31.8%), fast casual (25.9%), and specialty (4.7%), which included coffee shops and bakeries. They offered a variety of foods, including general American cuisine, sandwiches, pizza, and various ethnic cuisines. There were no significant differences in restaurant characteristics between more and less food secure neighborhoods.

**Table 1:** Characteristics of assessed restaurants by food security level.

Characteristics		Total (*n* = 85)	More food secure neighborhoods (*n* = 36)	Less food secure neighborhoods (*n* = 49)
		*n* (%)		
Type	Fast food	32 (37.6)	15 (41.7)	17 (34.7)
	Fast casual	22 (25.9)	11 (30.6)	11(22.4)
	Sit-down	27 (31.8)	10 (27.8)	17 (34.7)
	Specialty^a^	4 (4.7)	0 (0.0)	4 (8.1)
Cuisine	General/Mixed/American	18 (21.2)	8 (22.2)	10 (20.4)
	Sub Sandwiches	11 (12.9)	6 (16.7)	5 (10.2)
	Pizza	9 (10.6)	6 (16.7)	3 (6.1)
	Other^b^	45 (55.3)	16 (44.4)	29 (63.3)

^a^Specialty includes coffee shops and donut shops selling meals. ^b^Other includes BBQ, Italian, Asian, Chicken and other types of foods.

#### 3.1.1 Nutritional qualities of restaurant environments

The total nutritional quality scores of restaurants are shown with sub scores of availabilities of healthy foods, facilitators of healthy eating, and barriers of healthy eating in Table 2. The median of total nutritional quality scores was slightly higher at restaurants in more food secure neighborhoods (median = 5.5, IQR: 3–7.5) than those in less food secure neighborhoods (median = 4, IQR: 1–6.5) (*p* = 0.064). Specific scores of availabilities of healthful options, facilitators of healthy eating, and barriers of healthy eating did not show significant differences between neighborhoods.

**Table 2:** Nutritional qualities of assessed restaurant environments by food security level.

Categories	Total (*n* = 85)	More food secure neighborhoods (*n* = 36)	Less food secure neighborhoods (*n* = 49)
	Median (Interquartile Range)		
Total score (total range: −5 to 21)	5 (2–7)^*^	5.5 (3–7.5)	4 (1–6.5)
Availability of healthful options (range: 0 to 15)	4 (2–6.5)	4 (3–6)	4 (1–7)
Facilitators of healthy eating (range: 0 to 6)	0 (0–2)	1 (0–2)	0 (0–2)
Barriers of healthy eating (range: −5 to 0)	0 (0–1)	0 (0–0)	0 (0–1)

^*^ *p* < 0.1 for Kruskal-Wallis nonparametric comparison.

Individual assessment items were analyzed to see what specific items were provided by restaurants in each area. There was a tendency for restaurants in more food secure areas to provide more healthy options and better facilitate healthy eating. For example, a higher percentage of restaurants in more food secure areas offered healthy main-dish salad (50%), low-fat or fat free salad dressing (25%), and whole grain bread (28%), compared to those in less food secure areas (45, 14, and 22%, respectively). However, most items did not show statistically significant differences between the areas, except for two items. Healthy entrées were more available at restaurants in more food secure areas than in less secure areas (ꭓ^2^ = 3.417, df = 1, *p* = 0.065). Nearly 20% more restaurants in more food secure areas offered healthy entrées. Healthy eating was more actively promoted through signs, table tents, and displays at restaurants in more food secure areas than in less secure areas (ꭓ^2^ = 4.233, df = 1, *p* = 0.040). Eight percent of restaurants in more food secure areas displayed signs such as ‘Good greens, antioxidants,’ while no restaurants in less food secure areas displayed signs. Barriers to healthy eating such as encouraging large portions, unhealthy eating, or overeating were also not significantly different between the two areas, although there was a grater tendency toward these actions in less food secure areas. The difference in nutritional qualities of restaurant environments in more food secure versus less food secure neighborhoods (the first research question) is partially shown.

### 3.2 Dietary factors related to FAFH consumption

A total of 180 participants completed surveys that were used for comparisons of participants residing in more food secure neighborhoods (*n* = 39) and less food secure neighborhoods (*n* = 141). The average age of participants was 50.6 ± 15/1 years. Most participants were female (*n* = 127; 70.6%). The sample was somewhat racially diverse with participants identifying themselves as black being the largest group (*n* = 98; 54.4%) followed by participants identifying themselves as white (*n* = 53; 29.4%) being the second largest. A small percentage of participants identified themselves as Native American (*n* = 7; 3.9%), Asian (*n* = 4; 2.2%), or other (*n* = 11; 6.1%).

Mann–Whitney U tests revealed no significant difference in the total number of FAFH visits between residents of more food secure neighborhoods and less food secure neighborhoods (U = 3037.5; *p* = 0.254) was demonstrated, but a significant difference was identified between the more food secure neighborhood residents and less food secure neighborhood residents in fast food purchases (U = 3365.5; *p* = 0.012) (Table 3).

**Table 3:** Weekly frequency of food away from home purchases between residents from more food secure neighborhoods and less food secure neighborhoods.

Type of food away from home (Weekly)	Total (*n* = 180)	More food secure neighborhoods (*n* = 39)	Less food secure neighborhoods (*n* = 141)	*p*-value
	Mean ± Standard Deviation			
Total food away from home	2.75 ± 3.14	2.21 ± 2.45	2.90 ± 3.30	0.254
Fast Food	0.69 ± 1.04	0.39 ± 0.85	0.77 ± 1.01	0.012*
Full-service	0.54 ± 0.86	0.58 ± 0.72	0.53 ± 0.90	0.226
Takeout Fast Food	0.91 ± 1.16	0.072 ± 1.15	0.96 ± 1.16	0.110
Takeout full- service	0.63 ± 1.13	0.52 ± 0.73	0.66 ± 1.22	0.993

*p < 0.05 for Mann–Whitney U test.

Mann–Whitney U tests revealed no significant differences between the less food secure neighborhood residents and the more food secure neighborhood residents for the importance of taste (U = 3,048; *p* = 0.232), food safety (U = 2,690; *p* = 0.629), and nutrition (U = 2862.5; *p* = 0.159). A significant difference was demonstrated in the importance of food prices between participant groups (*p* = 0.034) (Table 4).

**Table 4:** Dietary factors related to food away from home consumption for residents from more food secure neighborhoods and less food secure neighborhoods.

Factors	Total (*n* = 180)	More food secure neighborhoods (*n* = 39)	Less food secure neighborhoods (*n* = 141)	*p*-value
	Mean ± Standard Deviation			
Taste	5.75 ± 1.80	5.51 ± 1.92	5.83 ± 1.76	0.232
Food Safety	6.05 ± 1.60	6.04 ± 1.45	6.04 ± 1.64	0.629
Food Price	5.68 ± 1.71	5.33 ± 1.59	5.78 ± 1.73^*^	0.034*
Nutrition	5.49 ± 1.61	5.32 ± 1.38	5.54 ± 1.67	0.159

^*^p < 0.05 for Mann–Whitney U test.

A significant association between food security groups was demonstrated when groups were asked if healthy food costs too much to eat. Residents living in less food secure neighborhoods were more likely to find healthy eating costs too much than those living in more food secure neighborhoods (ꭓ^2^ = 3.878, df = 1, *p* = 0.049). No significant associations were found when residents were asked if they were too busy to eat healthy food (ꭓ^2^ = 0.763, df = 1, *p* = 0.763) and when asked if healthy food does not taste good (ꭓ^2^ = 0.585, df = 1, *p* = 0.58). The difference in dietary factors related to FAFH consumption between more food secure and less food secure neighborhoods (the second research question) is partially shown.

## 4 Discussion

Current food assistance programs and policies primarily focus on food consumed at home. However, FAFH spending exceeds at home food spending across food secure and food insecure populations (1). To better understand the need for health policies and initiatives that target food away from home, this study investigated the nutritional qualities of restaurant environments and dietary factors related to FAFH consumption across different food security levels. The similarity in restaurant characteristics between more and less food secure neighborhoods in the studied county contrasts with previous findings of a higher density of fast food in food insecure areas (25). The finding suggests that fast food is generally more predominant throughout this county.

Previous research has reported mixed findings regarding the nutritional quality of restaurants by income level, showing either no significant differences across neighborhoods (26, 27) or a greater availability of healthier options in higher-income areas (28). Although the pattern of higher fast food density in less food secure areas is well established, caution is warranted when generalizing this pattern to the specific nutritional qualities of restaurants. Therefore, this study assessed the county food environment to help identify areas in need of policy development. Based on the results on the nutritional qualities of the restaurant environment, restaurants in more food secure neighborhoods provided slightly more nutritional qualities than those in less food secure neighborhoods, even though the differences were not substantial. A few restaurants in food secure neighborhoods also provided signage of healthy food options where none were found in the restaurants in less food secure neighborhoods. Difference in healthy food access as well as promotion of these items may exist that impacts individuals living in food insecure neighborhoods. This is concerning because previous research found a positive association between FAFH frequency and increased BMI for those experiencing food insecurity versus food secure individuals (2). Creating a FAFH environment that does not differ with respect to the type of neighborhood is needed. To do so, it may be necessary to develop policies that incentivize restaurants to provide food options and signage that do not differ based on restaurant location.

Nearly 70% of assessed restaurants, regardless of neighborhood type, offered a variety of healthy entrées-ranging from veggie options (e.g., veggie sandwiches and salads), grilled items (e.g., grilled salmon), and healthy beverages (e.g., diet soda, tea). This implies that restaurants are responding to growing consumer demand and awareness of vegetarian and health-conscious diets (29, 30). Despite availability of healthy entrees, there are still opportunities for improvement. The overall nutritional scores from the NEMS-R were low across all neighborhoods, ranging from 4 to 5.5 out of a possible 21. Restaurants offered fewer options for fruits without added sugar, non-fried vegetables, and whole grains. The consumption of these foods is strongly recommended due to health benefits such as reduced risk of weight gain, cardiovascular disease, type 2 diabetes, and cancer (31, 32). Restaurants need to take more active steps to improve the nutritional value of side items by incorporating more fruits, vegetables, and whole grains.

The rate of FAFH visits did not differ between the residents of the food secure and food insecure neighborhoods. The only difference in FAFH visits was that residents of food insecure neighborhoods frequented fast food restaurants more often. Interestingly, there were no differences in the factors related to FAFH consumption except that the residents in the less food secure neighborhoods believed healthy food cost more, but residents living across both more and less food secure neighborhoods felt nutrition was important when selecting food at a restaurant. Health is important to both groups, but cost may preclude individuals struggling with food insecurity to purchase less healthy FAFH options. Developing policies to not only promote the availability of healthy options while eating away from home but also provide mechanisms to provide healthier FAFH options that are not cost prohibitive.

Guidelines related to restaurant menus and labeling have the potential to help individuals find the nutritious meals they feel are important. Previous research has explored menu labeling changes to better guide consumers to select healthy choices (33–35). Policies like these are important in changing the overall FAFH food environment regardless of food security level and need to continue to be expanded. FAFH policy may need to become the primary focus to improve dietary intake instead of trying to change FAFH practices in individuals. For example, policy requiring calorie information on menus has been associated with reduced calorie intake (34). A concern though is that the use of nutrition information remains low among individuals with lower education levels and incomes (33). Consumers with limited health and functional literacy skills often struggle to understand and apply caloric values from menu labeling, thus a contextual or interpretive approach has been suggested (35). Again, this strengthens the argument of changing the overall FAFH environment through policy instead of the continued focus on educational strategies to reduce FAFH food consumption.

A limitation of this study is food insecurity was measured only by FII level based on residential zip code and not individually. Future research should also include a measurement within the survey that captures individual food security levels. While the findings of this study contribute to an increased understanding of FAFH in varying food secure neighborhoods, it should be considered that the samples sizes of the more food secure and less food secure groups were unbalanced; future studies should focus on methods to recruit more balanced samples to further strengthen these exploratory findings. While participants were recruited from a variety of locations, the high level of participation from food pantries contributed to this sample bias. The use of the validated NEMS-R tools and FII measurements that capture environmental-related metrics strengthens the validity and credibility of the study.

## 5 Conclusion

As demonstrated by the findings, individuals value having nutritious FAFH options, regardless of living in more or less food secure neighborhoods. Continued efforts by restaurants to reduce large portions and other obstacles to healthy eating remain essential in creating healthy food environments across both food secure and food insecure communities. While previous research has shown that restaurant managers generally support programs that promote healthy menu changes, one of their major concerns with implementing these changes is long-term consumer support and overall costs (36).

Since FAFH frequency varies by restaurant type, specifically fast-food purchases, future research should further investigate the nutritional qualities of restaurant environments and dietary factors related to FAFH consumption by restaurant type. It is also important for future research to explore cost effective changes restaurants can make to support healthy food options that appeal to consumers. Nationally, access to healthy FAFH options can be supported by expanding the SNAP Restaurant Meals Program to all states and SNAP eligible individuals (16). At the local level, there is a need for policymakers and community organizations to place greater emphasis on improving restaurant environments, while exploring ways to help food insecure individuals purchase healthy foods without worrying about affordability.

## Data Availability

The raw data supporting the conclusions of this article will be made available by the authors, without undue reservation.
