# Supplementary material for: Assessing restaurant nutrition quality and dietary factors that influence purchase of food away from home across different food security levels
Source: Front Public Health. 2026 Jan 23;14:1727510. doi: 10.3389/fpubh.2026.1727510 (PMC12875987; doi:10.3389/fpubh.2026.1727510)
Supplement: Supplementary file 1 [file Data_Sheet_1.PDF]

Do you live in Cuyahoga County?

Yes No

What is the zip code of where you reside?__________________

How often do you eat out at Fast Food restaurants in a typical week? Only include the times you DINE IN at the restaurant; do not include take-out or delivery.

__________________ Times per week

How often do you eat out at Full-Service restaurants in a typical week? Only include the times you DINE IN at the restaurant; do not include take-out or delivery.

__________________ Times per week

How often do you eat Fast Food through take-out or delivery in a typical week? Only include the times you TAKE-OUT or get DELIVERY; do not include dining in.

__________________ Times per week

How often do you eat Full-Service restaurant foods through take-out or delivery in a typical week? Only include the times you TAKE-OUT or get DELIVERY; do not include dining in.

__________________ Times per week

How important are the following attributes when selecting foods at restaurants?

	Not at all important	Low importance	Slightly important	Neutral	Moderately important	Very Important	Extremely Important
**Food Taste**	☐	☐	☐	☐	☐	☐	☐
**Food Safety**	☐	☐	☐	☐	☐	☐	☐
**Food Price**	☐	☐	☐	☐	☐	☐	☐
**Food Nutrition**	☐	☐	☐	☐	☐	☐	☐

Please circle the best choice for the statements below:

It costs too much for (me/my family) to eat healthy foods. Agree Disagree

I'm too busy to take the time to prepare healthy foods. Agree Disagree

I don't think healthy foods taste good. Agree Disagree

What is your age? (years old) _______________________

Which of the following best describes your gender?

- ☐ Woman
- ☐ Man
- ☐ Non-binary
- ☐ Prefer to describe (*please describe your gender*) _________________
- ☐ Prefer not to answer

What is your monthly household income? (dollars) _$_________________________

Are you Hispanic or Latino/a?

- ☐ Yes
- ☐ No
- ☐ Prefer not to answer

Which of the following best describes your race or ethnicity? (please check all that apply)

- ☐ American Indian or Alaska Native
- ☐ Asian
- ☐ Black or African American
- ☐ Native Hawaiian or Pacific Islander
- ☐ White
- ☐ Some other race, ethnicity, or origin (*please describe*) _________________
- ☐ Prefer not to answer

What is the highest level of education you have completed?

- ☐ Some high school or less, but no diploma
- ☐ High school diploma or equivalent
- ☐ Some college or associate degree
- ☐ Bachelor's degree
- ☐ Graduate degree (e.g., master's, doctorate, or professional degree)

Thank you for completing the survey! If you'd like to receive a $5 Amazon gift card through email, please share your email address with the student researchers when you return your survey.
